# Dr. Goudret’s Ammoniacal Blistering Ointment

**Published:** 1846-06

**Authors:** 


*Dr. Goudret's Ammoniacal Blistering Ointment.*—The following improved formula for this application is recommended by the author in preference to that which has hitherto been in use:

| | | |
|---|---|---|
| Take of lard | - - - - - - | 32 parts. |
| " Oil of almonds | - - - - | 2 " |

Melt the lard with the oil by the application of a gentle heat: pour them in the melted state into a wide-mouth bottle, and add

| | | |
|---|---|---|
| Solution of ammonia | - - - | 17 parts |

Mix, by continual agitation, until it becomes cold. It is necessary to avoid the application of much heat in the preparation of this ointment. When well prepared it will produce vesication in about ten minutes, and will retain its properties unimpaired for about a month, if kept in a well-stopped bottle.—*Dub. Med. Press, from Journal de Pharmacie.*

